# Effect of Yd:YAG laser irradiation on the shear bond strength of orthodontic metal brackets

**DOI:** 10.1590/2177-6709.25.1.028-035.oar

**Published:** 2020

**Authors:** Fernando César Moreira, Helder Baldi Jacob, Luis Geraldo Vaz, Antonio Carlos Guastaldi

**Affiliations:** 1 Universidade Estadual Paulista, Faculdade de Odontologia, Departamento de Materiais Odontológicos e Prótese (Araraquara/SP, Brazil).; 2 The University of Texas Health Science Center at Houston School of Dentistry, Department of Orthodontics (Houston/TX, USA).; 3 Universidade Estadual Paulista, Instituto de Química, Departamento de Físico-Química (Araraquara/SP, Brazil).

**Keywords:** Orthodontic brackets, Laser ablation, Shear strength, Orthodontic adhesives, Dental enamel

## Abstract

**Objective:**

The purpose of this study was to evaluate the effect of the Yd:YAG laser irradiation on orthodontic bracket base surface. Shear bond strength (SBS) values and sites of the bonding failure interfaces were quantified.

**Methods:**

Brackets were divided into two groups: OP (One Piece - integral sandblast base) and OPL (One Piece - laser irradiation). The brackets were randomly bonded on an intact enamel surface of 40 bovine incisors. The SBS tests were carry out using a universal test machine. A stereomicroscopy was used to evaluate the adhesive remnant index (ARI), and surface characterization was performed by scanning electron microscopy (SEM). Student’s t-test was used to compare the SBS between the two groups (*p*< 0.05). Frequencies and chi-square analysis were applied to evaluate the ARI scores.

**Results:**

OPL group showed higher value (*p*< 0.001) of SBS than OP group (43.95 MPa and 34.81 MPa, respectively). ARI showed significant difference (*p*< 0.001) between OPL group (ARI 0 = 100%) and OP group (ARI 0 = 15%). SEM showed a higher affinity between the adhesive and the irradiated laser base surface.

**Conclusions:**

Yd:YAG laser irradiation on bracket base increased SBS values, showing that bonding failure occurs at the enamel/adhesive interface. Laser-etched bracket base may be used instead of conventional bases in cases where higher adhesion is required, reducing bracket-bonding failure.

## RESUMO

**Objetivo:**

O objetivo do presente estudo foi avaliar o efeito da aplicação do *laser* Yd:YAG sobre a superfície metálica da base de braquetes ortodônticos, a resistência da colagem ao cisalhamento (RU) e os locais das falhas nas interfaces adesivas.

**Métodos:**

Os braquetes foram divididos em dois grupos, de acordo com o tipo de base: OP (One Piece - monobloco, base jateada) e OPL (One Piece - monobloco, base irradiada a *laser*). Foram selecionados 40 incisivos bovinos hígidos para a colagem de braquetes na superfície do esmalte. Os testes de RU foram realizados por meio de uma máquina de ensaio universal EMIC DL1000. O Índice de Adesivo Remanescente (ARI) e a caracterização das superfícies foram realizados empregando-se estereomicroscópio e microscópio eletrônico de varredura (MEV). O teste *t* de Student foi aplicado para comparar a RU entre os dois grupos (*p*< 0,05). O ARI foi avaliado por meio da análise de Frequência e teste do Qui-quadrado.

**Resultados:**

O grupo OPL obteve maiores valores de RU (*p*<0,001) do que o grupo OP (43,95 MPa e 34,81 MPa, respectivamente), e o ARI obteve diferença significativa (*p*<0,001) entre os grupos OPL (ARI 0 = 100%) e OP (ARI 0 = 15%). A análise proveniente do MEV mostrou alta interação adesiva entre a resina e a superfície metálica da base irradiada pelo feixe de *laser*.

**Conclusões:**

A irradiação com *laser* Yd:YAG sobre a superfície da base de braquetes metálicos aumentou os valores da RU, demonstrando que as falhas adesivas ocorreram na interface esmalte/adesivo. O uso de braquetes modificados com irradiação a *laser* pode ser adotado como alternativa aos braquetes com bases convencionais nos casos em que uma maior retentividade for requerida para reduzir as falhas de adesividade.

## INTRODUCTION

During orthodontic treatment, bracket adhesion to enamel, through the adhesive system, should be high enough to resist failure^1^ ^-^ ^3^ but also permit removal after treatment without causing any enamel damage.^1^ ^,^ ^2^ The failure of the bracket adhesion to the enamel can delay treatment completion, increasing the costs relative to the maintenance of the fixed orthodontic appliances. 

Factors affecting the bracket bond strength on enamel have been extensively studied, including: specimen storage time, enamel conditioning procedures, masticatory forces, adhesive systems, and bracket-related factors (such as size, structure of the mesh, and material of the bracket base).^1^ ^,^ ^4^ ^-^ ^8^ Previous studies reported that the bond failure of the metal bracket to tooth enamel frequently occurs at the adhesive/bracket base interface.^2^ ^,^ ^3^ ^,^ ^5^ ^,^ ^8^ ^-^ ^12^

Bracket base design plays an important role in terms of the bond strength to the tooth enamel.^2^ ^,^ ^5^ ^-^ ^7^ ^,^ ^9^ ^,^ ^13^ ^,^ ^14^ Because metal brackets do not chemically adhere to enamel or resin, studies have been performed to improve the mechanical retention.^5^ Modifications in the bracket base design have been made to improve the micro-interlocking mechanism at the bracket/adhesive interface, to achieve satisfactory bond strength.^2^ ^,^ ^7^ ^,^ ^10^ Several chemical treatment and mechanical retentive designs or a combination of both systems have been accomplished in order to enhance the retention of the adhesive to the metal base of orthodontic brackets.^7^ ^,^ ^12^ ^-^ ^14^

Laser treatment is an innovative method that increases specific surface area, and enhances wettability and surface energy^15^. This technique produces micro and nanostructure material property changes by physicochemical modification that creates desired surface patterns. The laser is scanned over the base surface, melting and solidifying the metal, to create irregularities and microporosities on the bracket base creating retentions for the adhesive..^7^ ^,^ ^8^

To the date, there is no study comparing the same metal bracket base design with and without laser irradiation (Yd:YAG laser). This study was designed based on the idea that bracket base surface modified by laser could increase bond strength. The aim of this study was to evaluate the effect of the application of the Yd:YAG laser on the orthodontic metal bracket base surface. Shear bond strength (SBS) and sites of bonding failure at the bracket/adhesive/enamel interfaces were measured. The null hypothesis of this study was that there are no significant differences in SBS values and sites of the debonding between sandblasted bracket bases and laser-etched bracket bases.

## MATERIAL AND METHODS

### Bracket preparation

**Figure 1:**
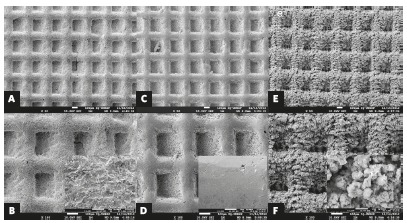
Scanning electron microscope analysis of brackets: A, B) One Piece - sandblasted base; C, D) One Piece without sandblasting; E, F) One Piece after laser-etching. (SEM, 50x, 100x, 2.000x).

Two groups (n = 20) were created according to their bracket type design: OP (One Piece bracket) and OPL (One Piece bracket with laser irradiation). The One Piece brackets (Aditek Orthodontics, Cravinhos, Brazil) are manufactured using a metal injection molding and present 80-gauge integral sandblasted mesh base with 10.92 mm^2^ surface area (Fig 1). Using Fiber Optic OmniMark 20F (OmniTek, São Paulo, Brazil) the OPL group received pulsed Yd:YAG laser irradiation on the bracket base surface (Fig 1). The output power of the laser was 100% and the speed laser parameter that describes the movement of the laser head was ranging from 300 to 500 m/s. The frequency parameter ranged from 5 to 35 kHz, and fluence was 19 J/mm^2^. All the samples were cleaned by ultrasonic method (UltraSonic Clear, USC 1450 model, Unique Ind e Com, São Paulo, Brazil) using ethanol 96% and distilled water during 5 minutes before the bonding.

### Teeth and bonding procedures

Forty bovine incisors with intact labial surface and no defects on the enamel were selected. After removing any debris, the roots were partially sectioned. The specimens were vertically positioned using a customized positioner device into PVC cylinders filled up with self-cured acrylic resin (VIPI Flash, Pirassununga, SP, Brazil) until approximately 2 mm of cement-enamel junction was exposed. All teeth crowns were polished with a rubber cup and pumice paste at low speed for 10 seconds, and rinsed with water spray for the same time, air-jet dried, and stored in distilled water at 37 °C. Then, enamel surface was etched with 37% phosphoric acid (Condac 37, FGM, Joinville, SC, Brazil) for 30 seconds, rinsed with water spray for 15 seconds, and dried with oil-free compressed air for the same time. Transbond^™^ XT (3M Unitek^™^, St. Paul, MN) primer-adhesive was applied on the etched surface, followed by a light air jet to complete flow, and it was polymerized by LED Bluephase (Ivoclar Vivadent, Liechtenstein, Austria) for 10 seconds right away. 

**Figure 2:**
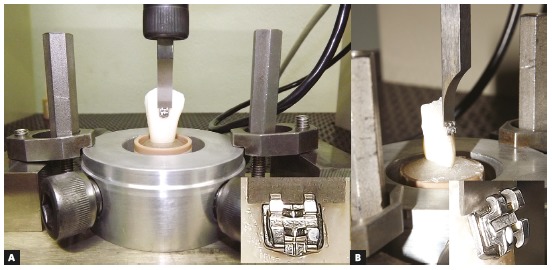
Specimen positioned in the loading rig at the mechanical test machine: A) Frontal view and B) lateral view with close-up of the shearing blade at the enamel/bracket interface, during the SBS test.

At the bracket, Transbond^™^ XT primer (3M Unitek^™^, St. Paul, MN) was applied on the bracket base surface followed by a gentle air jet. This procedure allows the primer to flow inside the bracket base micro-retentions, obtaining a wetting surface of adhesive, in order to increase mechanical retention of the adhesive to the pad.^16^ Then, a tensiometer device (Morelli, Sorocaba, SP, Brazil) applying 300 gf compression was used for positioning the brackets.^16^ Resin excess was removed from the margin of the bracket with a dental probe before setting. Resin polymerization was accomplished with a LED Bluephase (Ivoclar-Vivadent, Liechtenstein, Austria) on each side of the bracket base for 20 seconds, with intensity around 1200 mW/cm² at an approximated distance of 3 mm. After bonding, the samples were incubated in a 37 °C water bath for 24 hours. These procedures were performed for all samples. The SBS tests were accomplished by using a universal test machine EMIC DL1000 (EMIC Equipamentos e Sistemas de Ensaio Ltda, São José dos Pinhais, PR, Brazil). Each specimen was positioned in the loading rig with its labial surface parallel to the force during the SBS tests. An occlusogingival load was applied to each sample, producing a shear force at the enamel/bracket interface using a shearing blade (6.0 mm*x*0.4 mm) at a speed of 0.5 mm/min and 100 kgf load cell (Fig 2). A computer coupled to the universal test machine recorded the outcomes in megapascals (MPa) for each test.

A stereomicroscope (Leica M80, Leica Microsystems, Wetzlar, Germany) was used to examine each sample at 20x magnification. According to the remaining amount of composite on the enamel surface, after shear tests, an Adhesive Remnant Index (ARI) was used.^17^ The scores were quantified as: 0 = no bonding resin left on the tooth; 1 = less than half of the bonding resin left on the tooth; 2 = more than half of the bonding resin left on the tooth; and 3 = all bonding resin left on the tooth, with a distinct impression of the bracket mesh^17^. Micrographs were obtained from each group before and after the shear tests, using a scanning electron microscopy (JSM-7500F, JEOL Ltd, Tokyo, Japan), in order to perform surface characterization, analyze the bonding interfaces and failure sites.

### Statistical analysis

Mean SBS values and standard deviations of samples were performed using IBM SPSS^™^ statistics (version 23 for Windows, SPSS, Armonk, NY). Normality test was done using the Shapiro-Wilk test, and the data were normally distributed. Student’s t-test was applied for overall comparison of groups, to determine the effect of bracket base design on mean SBS. Frequencies and chi-square analysis were used to determine significant differences in ARI scores between groups. Statistical significance was determined at *p*< 0.05. The sample size of this study was not calculated *a priori*. Based on sample size, mean, and standard deviation of the two groups, with a large effect size (f=0.848), this study had a power of 0.743.

## RESULTS

### Shear bond strength

OPL group showed higher shear bond strength than OP group (43.95 MPa and 34.81 MPa, respectively) (Table 1). There was a significant effect of Yd:YAG laser irradiation on SBS, at the *p*< 0.05 level for the three conditions [F(1.38) = 2.566, *p*= 0.014].

**Table 1:** Descriptive statistics and statistical comparisons for SBS (MPa) between OP and OPL groups

Groups	Min-max	Mean	SD	SEM	Variances	p-value
OP	17.20-55.40	34.81	12.72	2.84	161.71	0.228 (F)
OPL	29.40-63.00	43.95	9.60	2.15	92.10	*0.014 (t)*

SD: Standard deviation; SEM: Standard error mean; p-value, F-test and t-test. Bold-italic mean statistically significant at p < 0.05.

### Adhesive remnant index (ARI)

OPL group showed ARI scores of 0 (100% of the specimens), indicating that none of the adhesive remained on the surface of the enamel and all of the composite adhered to the bracket base surface (Table 2), while OP group obtained varied results. When the groups were combined, the majority of fractures (57.50%) occurred at the enamel/adhesive interface (ARI = 0). Statistical analysis exposed significant differences within the samples, ARI scores were significantly different between the two groups [χ^2^(3) = 29.565, *p*< 0.001].

**Table 2:** Distribution frequency, percentages of ARI scores, and statistical comparison between groups.

ARI scores	OP	OPL	χ**2**	Gl*	Prob
0	3 (15%)	20 (100%)			
1	4 (20%)	0	29.565	3	<0.001
2	7 (35%)	0			
3	6 (30%)	0			

Χ^2^: Chi-Square; *Df: Degrees of freedom; Prob.: probability (statistically significant at p < 0.05).

### Scanning electron microscopy (SEM)

**Figure 3:**
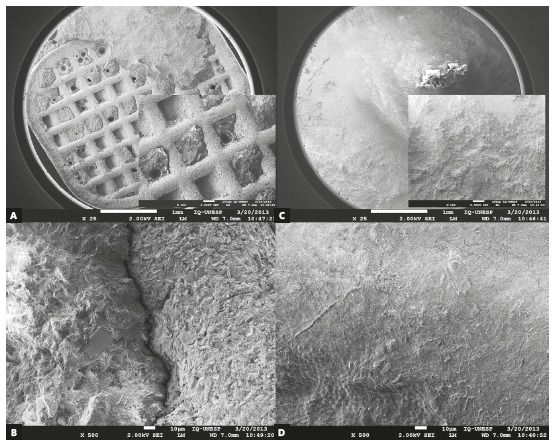
Scanning electron microscope analysis of bracket bases after debonding: A, B) One Piece - sandblasting base; C, D) One Piece after laser etching. (SEM, 25x, 100x, 500x).

Using SEM for comparative surface topography analysis among the studied bracket bases before and after shear bond tests, it was clearly noticed that OPL group retained the entire amount of composite on the bracket base (ARI = 0, 100%) while the frequency distribution of ARI scores varied significantly in the OP group (Fig 3). The quantity of adhesive remained on the base in the OP group covered about half the area of the base of the brackets in 55% of the samples, and only 15% of the specimens presented ARI = 0. Under stereomicroscopy at 20x magnification, no enamel damage was noticed.

## DISCUSSION

Laser irradiation applied on the bracket surface increases shear bond strength. The results clearly indicates the influence of the Yd:YAG laser in the retention mechanism. When the same type of bracket received laser irradiation on its base, the SBS increased approximately 26.5%. Some previous studies have reported a higher shear bond strength when the laser was used in the manufacture process of the bracket base.^6^ ^,^ ^18^ ^-^ ^20^ On the other hand, it also has been shown no significant difference^21^ or even lower SBS between laser structured bracket base and another type of bracket base.^6^ The inconsistencies in the literature are mainly due to study designs (i.e. different bracket base designs or different cross-sections among the bracket bases).^22^ The present study was the first to compare the SBS using, among the bracket bases tested, the same bracket with and without Yd:YAG laser-structured bracket base. The highest SBS can be explained by changes in the topography of the bracket base produced by laser irradiation, resulting in composition changes of the irradiated material.^15^

Different bracket base treatment leads to different strength adhesion to the enamel. The literature has shown that size and design of the bracket base affect the bond strength to the enamel.^4^ ^,^ ^5^ ^,^ ^14^ ^,^ ^20^ ^,^ ^23^ ^,^ ^6^ ^-^ ^13^ Altmann et al.^4^ showed that bracket base area is an important variable in SBS. Also, it is known that the bracket base curvature should adapt to the tooth surface, allowing small thickness of resin between the base and the enamel, otherwise it could result in a weak interface due to the inherent mechanical properties of the resin.^4^ Finite element analysis provided a clearer insight of the stress distribution and showed that the greater bracket base adaptation on the tooth surface, the distribution of forces along its surface will be more uniform.^24^ In addition, laser parameters such as power, wavelength, frequency, scanning speed and the exposure of the bracket surface area, can promote different outcomes for SBS. Because OP group and OPL group presented the same bracket design and size, the highest SBS obtained by the OPL group can be explained due to the micro-retentions produced at the metallic base surface by the ablation process. 

The use of laser-etched bracket base changes considerably the interface failure between bracket and enamel. OPL group showed a strong mechanical bond between the laser-etched base and the adhesive. No other *in vitro* study showed such high adhesion between bracket and bonding agent. Evaluating different bracket bases, some authors reported up to 80% of the adhesive resin remaining on the laser-structured base,^7^ ^,^ ^20^ ^,^ ^21^ and Rajesh et al.^8^ in their research found 60% ARI = 0. Interestingly, Cozza et al.^6^ showed that only 20% of fracture occurred at bracket-adhesive interface when laser-etched base was evaluated. Reports in the literature claims that the ARI score was dependent upon many factors, including the adhesive type and bracket base design.^2^ ^,^ ^6^ ^,^ ^8^ ^,^ ^11^ ^,^ ^12^ ^,^ ^20^

Bovine incisors were used in the present study due to the numerous advantages related to human teeth, being widely used in *in vitro* studies. Yassen et al.^25^ carried out a review to verify the use of bovine teeth as a substitute for human teeth, and found evidence that there were few differences between them, most associated to chemical properties and composition. High bond strength values are potentially dangerous to enamel and they could cause enamel fracture during debonding.^1^ ^,^ ^3^ ^,^ ^7^ ^,^ ^11^ ^,^ ^19^ ^,^ ^20^ Although shear bond strength test differs from tensile bond strength, the literature has referenced the potential enamel damage to tensile bond strength as low as 9.7 MPa.^11^ ^,^ ^26^ ^,^ ^27^ However, authors agree that the values of the SBS obtained *in vitro* are above the acceptable values *in vivo* and could carry an increased risk of enamel fracture.^20^ ^,^ ^28^ It is important to note that in clinical conditions brackets are exposed to varied intraoral factors (including different levels of tension) present at *in vivo* environment and must not be compared with the SBS tests performed under laboratorial conditions with a unit tooth factor. Several studies evaluating shear bond strength showed values up to 31.0 MPa, supporting the values obtained in the present study.^20^ ^,^ ^29^ ^,^ ^30^ Hofmann et al.^20^ found that the combination of Transbond^™^ XT with laser structured metal bracket base involved enamel fractures in 73% of the group samples;^20^ although all two groups in this study presented mean SBS values above 31.0 MPa, none enamel damage was observed in any group under stereomicroscopy. According to Elsaka et al.,^11^ although the enamel resist to the high level of forces showed in this study, orthodontic brackets debonding should be performed with care. In this sense, it is safe not to damage the dental enamel at the end of the orthodontic treatment.^1^ ^-^ ^3^ ^,^ ^7^ ^,^ ^11^ ^,^ ^14^ ^,^ ^18^ ^,^ ^20^ ^,^ ^21^ ^,^ ^29^ The SBS values of the OPL group (between 29.0 and 63.0 MPa) in present study are in accordance with others authors,^20^ ^,^ ^25^ but results should be viewed with discretion regarding whether bovine teeth could be considered appropriate to substitute human teeth despite their similar structures. Therefore, all results obtained in this *in vitro* study must be considered just as an estimate to clinical outcomes and should be compared with others researches results performed on bovine teeth.

The laser-etched bracket base increases the amount of bonding agent retained at the bracket pad. The laser-treated bracket base allowed a greater physical and chemical interaction (wettability and surface energy)^15^ between the biomaterial (resin/metal), resulting in 100% ARI score. The bracket base treated with Yd:YAG laser process increased almost seven times (ARI=0, OP 15%; OPL 100%) the amount of resin that was retained in the bracket pad, when compared to the same type of bracket without Yd:YAG laser process (Table 2). The differences between the groups can be attributed to the high-energy surface between the modified metal base by laser-beam irradiation^15^ and the resin adhesive, allowing a higher physical interaction between the interface bracket base/adhesive/resin than the resin/enamel. Present findings are in disagreement with Elsaka et al,^11^ which showed that metallic brackets have a tendency to fail mainly at the bracket/adhesive interface. Although testing different adhesive systems, Hellak et al^19^ showed an ARI = 0 in 65% or greater, depending of the adhesive system. Hoffmann et al^20^ found 85% of ARI = 0 at laser-structured metal bracket base, when compared to other base designs and different adhesive systems combinations. Also, the authors showed that Tranbond^™^ XT presented only 25% of ARI = 0 when different adhesive systems were tested. Adhesive remnant index values are subjective and should be interpreted with caution, and the three-dimensional surface and retention system of the bracket base is frequently ignored.^2^ Also, variation of the base design and amount of the adhesive may result in different ARIs, which could affect the enamel surface during debonding.^2^

The use of laser-structured bracket base retention might be more than sufficient to obtain clinically optimal bond strength. Although bracket base after laser irradiation presented high SBS, no damage to the enamel surface was reported. Some studies reported less adhesive remaining on the enamel surface with high adhesion.^2^ ^,^ ^7^ ^,^ ^8^ ^,^ ^19^ ^,^ ^22^ Another advantage has been found by eliminating chemical or silanization methods.^7^ ^,^ ^8^ In this context, laser irradiation has been suggested as an alternative technique for biomaterial industry.^15^ Therefore laser-etching technology is an innovative approach, resulting in increased surface area, enhanced wettability, and increased bond strength of the orthodontic metal brackets to enamel. It could also be applied on dental treatment of non-cooperative patients, brackets to posterior teeth, and in other regions where increasing bond strengths are required.

## CONCLUSIONS

Based on the present results, the following conclusions can be drawn:

» Differences between OP and OPL groups were reported, rejecting the null hypothesis.
» The laser-irradiated base of the metal brackets increases the SBS value.
» Yd:YAG laser irradiation on the bracket base is a viable process to produce retentive bases and possibly allows smaller bracket bases.
» The laser-etched bracket base retained significantly more adhesive on bracket base.
